# Sir Joseph Beacham

**Published:** 1916-11

**Authors:** 


**Sir Joseph Beacham,** maker of the well known pills established by his father, the late Sir Thomas Beacham, died Oct. 23 aged 69.

